# Decoding the whole-genome sequence of multidrug-resistant *Escherichia coli* strain Hakim RU_BHWS isolated from wastewater in Bangladesh

**DOI:** 10.1128/mra.01069-24

**Published:** 2024-11-22

**Authors:** Md. Shamsul Islam, Muhib Ullah Khan, Nusrat Zahan, K. M. Golam Saklain, Miaomiao Liu, Masaaki Kitajima, Md. Hakimul Haque

**Affiliations:** 1 Department of Veterinary and Animal Sciences, University of Rajshahi 118869 , Rajshahi, Bangladesh; 2 Research Center for Water Environment Technology, School of Engineering, The University of Tokyo, Tokyo, Japan; 3 Biomedical Sciences & Molecular Biology, College of Public Health, Medical and Veterinary Sciences, James Cook University, Townsville, Queensland, Australia; University of Maryland School of Medicine, Baltimore, Maryland, USA

**Keywords:** whole genome, wastewater, multidrug-resistant, *Escherichia coli*, Bangladesh

## Abstract

This report presents the draft genome sequence of the multidrug-resistant *Escherichia coli* strain Hakim RU_BHWS isolated from wastewater. The genome assembly is 4.6 Mb, with 32.16× coverage and a GC content of 50.7%. It includes five CRISPR arrays, 16 prophages, 56 antibiotic resistance genes, and 35 virulence factor genes.

## ANNOUNCEMENT

In resource-limited countries, the discharge of wastewater-derived *E. coli* into the environment poses significant public health risks (1, 2) due to the spreading of the antimicrobial resistance (AMR) genes between pathogenic and environmental bacteria through horizontal gene transfer (3). The presence of multidrug-resistant *E. coli* in wastewater and the receiving environment underscores the need for AMR surveillance, which can inform targeted public health interventions to address this growing threat (4–7).

In September 2023, following approval from the Institute of Biological Sciences (IBScs) at the University of Rajshahi, Bangladesh (Memo No. 56/321/IAMEBBC/IBScs), we collected wastewater samples from the canal that received wastewater from the boys’ residential hall in the university (24.3733°N; 88.6049°E) using standard procedures. The samples were thoroughly mixed, placed into sterile tubes, and transported to the laboratory (7). *Escherichia coli* was isolated by inoculating the samples on MacConkey (HiMedia, India) and eosin methylene blue agar (HiMedia, India) and incubating them aerobically at 37°C for 18–24 hours, followed by staining and biochemical tests (8). The antimicrobial susceptibility profiles of the isolates were determined against thirteen commonly used antibiotics using the disk diffusion method (9), in accordance with Clinical and Laboratory Standards Institute (CLSI) guidelines (10). The multidrug-resistant strain exhibiting resistance to amoxicillin + clavulanic acid, tetracycline, doxycycline, cephradine, ciprofloxacin, co-trimoxazole, and azithromycin was subjected to genome characterization.

The strain was cultured overnight in nutrient broth (HiMedia, India) at 37°C, and genomic DNA was extracted using the Qiagen DNA Mini Kit (QIAGEN, Hilden, Germany). Genomic DNA was enzymatically fragmented with the NEBNext dsDNA Fragmentase Kit (NEB, MA, USA), followed by size selection using SPRI beads (11). A sequencing library was prepared with the Nextera DNA Flex Library Preparation Kit (Illumina, San Diego, CA, USA) and sequenced with 2 × 150 bp paired-end reads on the Illumina NextSeq2000 platform. Quality control was performed using FastQC v0.11.7 (12), and the raw reads (*n* = 8,408,03) were trimmed with Trimmomatic v0.39 (13). Genome assembly was performed using Unicycler v0.4.9 (14), and the annotation was conducted with PGAP v3.0 (15). Further analysis included antibiotic resistance gene detection with CARD v3.2.4 (16) and RGI v6.0.2 (17), virulence factor identification using VFDB and VFanalyzer v4.0 (18), pathogenicity index using PathogenFinder v.1.1 (19), sequence type using MLST v.2.0 (20), CRISPR arrays using CRISPRimmunity (21), and metabolic functional features using RAST v.2.0 (22). We used default parameters for all tools, unless noted otherwise.

**Fig 1:**
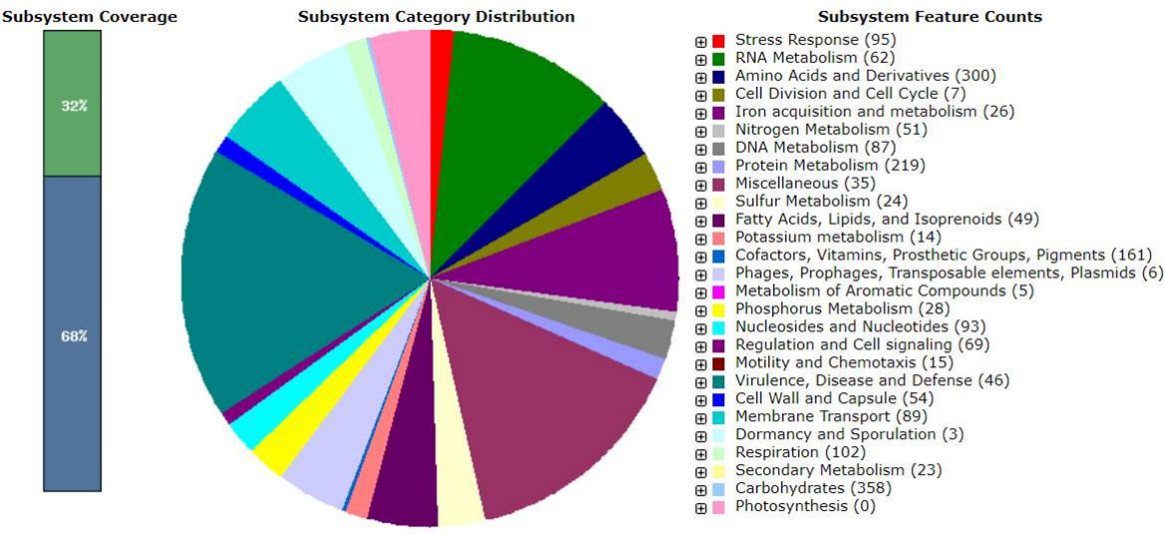
Metabolic functional features in the *E. coli* Hakim RU_BHWS assembled genome in SEED viewer. The 32% coverage indicates the completeness of functional roles within a specific subsystem across different genomes.

The components of the draft genomes are recorded in Table 1. Notably, 56 ARGs, including quinolone, folate pathway antagonist, and tetracycline antibiotic classes; 35 virulence genes comprising *yehD, terC, nlpl, kpsE, csgA, gad, AslA, hlyE, kpsM_K15, fdeC, ompT,* and *fimH;* and one plasmid [p0111] on contig 69, were deduced. Multilocus sequence typing (MLST) classified the genome as sequence type 84, and the PathogenFinder tool specified a pathogenicity index of 0.939. The genome exhibited five CRISPR arrays with 17 signature genes (*TnsC, csa3, cas2, cas1, cas6e, cas5, cas7, cse2gr11, cas8e, cas3, WYL, DEDDh, PD-DExK, DinG, RT, cas14i,* and *c2c9_V-U4*) and 16 prophages. RAST analysis uncovered 376 subsystems comprising 4,541 genes with 32% coverage (Fig. 1).

**TABLE 1:** Genomic components of the *E. coli* strain Hakim RU_BHWS

Components	Values
Genome size	4,639,095 bp
Genome coverage	32.16×
G + C content	50.7%
Number of contigs	108
Contig L50	15
Contig N50	92,850 bp
Total genes	4,545
Coding sequences	4,461
Coding genes	4,283
RNA genes	84
tRNA genes	73
rRNA genes	3
tmRNA genes	1
ncRNA genes	10
Pseudo-genes	178
Genes assigned to SEED subsystems	4,541
Number of subsystems	376

## Data Availability

The study on E. coli strain Hakim RU_BHWS, conducted using the WGS shotgun approach, was submitted to NCBI/GenBank, and the assembly was deposited under the accession number JBEHGO000000000. The pertinent data, including the original readings, were stored with BioProject accession number PRJNA1102289, BioSample accession number SAMN41016666, and SRA accession number SRR28743347. The specific version mentioned in this document is labeled as JBEHGO000000000.1.
